# Comparison of atlantoaxial and lumbosacral cerebrospinal fluid centesis techniques in South American camelids

**DOI:** 10.1111/jvim.17023

**Published:** 2024-02-26

**Authors:** Ester Malmström, Robert C. Cole, Erik H. Hofmeister, Jere K. Stern, Thomas Passler

**Affiliations:** ^1^ Department of Forestry and Wildlife Management Inland Norway University of Applied Sciences Evenstad Norway; ^2^ Department of Clinical Sciences, JT Vaughan Large Animal Teaching Hospital, College of Veterinary Medicine Auburn University Auburn Alabama USA; ^3^ Department of Pathobiology, College of Veterinary Medicine Auburn University Auburn Alabama USA

**Keywords:** alpaca, CSF, farm animal medicine, llama, ultrasound, veterinary diagnostics

## Abstract

**Background:**

Iatrogenic blood contamination during cerebrospinal fluid (CSF) centesis is common, which can limit the diagnostic usefulness of the sample. A novel ultrasound‐guided CSF collection technique is described in horses, by which CSF is obtained from the atlantoaxial (AA) space.

**Hypothesis/Objectives:**

To compare ultrasound‐guided AA centesis with lumbosacral (LS) centesis in South American camelids (SAC). The hypotheses were that AA centesis would yield samples with less blood contamination although being technically more challenging than LS centesis.

**Animals:**

Eight clinically healthy adult SAC from a university‐owned teaching herd.

**Methods:**

Single‐blinded, randomized, 4‐way, 4‐period crossover study in which 2 veterinarians each performed both centesis techniques on each animal once. Cytological sample analysis was performed, and the technical difficulty of sample acquisition was assessed.

**Results:**

The CSF was collected successfully and without complications by either technique during all collection attempts. Aspects of technical difficulty and concentrations of CSF analytes did not vary significantly between techniques. Median total nucleated cell and red blood cell counts were 1/μL and 0.5/μL and 167.5/μL and 155/μL for AA and LS techniques, respectively. The median total protein concentration was 32.9 mg/dL and 38 mg/dL for AA and LS centeses. A median of 1 attempt was necessary for both centesis techniques and the median number of needle repositioning events was 1 for AA and 0 for LS.

**Conclusion and Clinical Importance:**

Depending on clinical circumstances, ultrasound‐guided AA centesis appears to be an acceptable alternative to other techniques for collection of CSF from SAC.

**Abbreviations**
AA: atlantoaxial
AO: atlantooccipital
BW: body weight
CSF: cerebrospinal fluid
LS: lumbosacral
RBC: red blood cell
RBCC: red blood cell count
TNCC: total nucleated cell count
TPC: total protein concentration
SAC: South American camelid

## 1 INTRODUCTION

Clinicopathologic analysis of cerebrospinal fluid (CSF) is a valuable tool for establishing the diagnosis, treatment protocol, and prognosis in animals with central nervous system (CNS) disease.^1^, ^2^ A normal CSF sample is free of red blood cells (RBC).^3^ The presence of RBC can indicate CNS trauma, but is more commonly caused by iatrogenic blood contamination.^4^, ^5^ The diagnostic usefulness of a CSF sample can be limited by blood contamination causing artifactual increases in the total protein concentration (TPC) and total nucleated cell counts (TNCC).^5^, ^6^ Currently, CSF centeses in South American camelids (SAC) and other farm animal species are performed either at the atlantooccipital (AO) space or the lumbosacral (LS) space.^7^, ^8^ Centesis at the AO space has greater risks in comparison with LS centesis, because of needle placement close to the brainstem, requiring the animal to be under general anesthesia.^9^, ^10^

Occasionally, CSF samples can be difficult to obtain. Over the past 3 decades, ultrasonographic techniques consequently have been developed to aid with successful CSF collection in companion animals,^11^ cattle,^7^ small ruminants,^12^ and horses.^13^ A novel ultrasound‐guided CSF centesis technique has been described in horses, by which a CSF sample is obtained from the atlantoaxial (AA) space from standing sedated animals.^13^, ^14^ The sample is collected using a lateral approach while ultrasonographically visualizing the subarachnoid space between the atlas and axis vertebrae (C1‐C2). Despite the promising results of this collection technique in horses, ultrasound‐guided CSF centesis from the AA space has not been scientifically evaluated in SAC.

Our specific objectives were to: (a) describe the ultrasound‐guided AA CSF centesis technique in sedated sternally recumbent SAC and (b) compare the technical difficulty and CSF sample characteristics between the AA and LS centesis techniques. The research hypotheses were that the AA CSF centesis technique would yield samples with less RBC contamination compared with the LS centesis technique but would be more technically challenging.

## 2 MATERIALS AND METHODS

### 2.1 Animals

The study was approved by the Auburn University Institutional Animal Care and Use Committee (PRN 2021‐3837), and the animals were cared for according to the principles and guidelines set by the committee. The sample population consisted of 8 university‐owned, adult, healthy SAC (n = 2 female llamas, n = 1 intact male alpaca, n = 1 castrated male alpaca, n = 1 spayed female alpaca, and n = 3 intact female alpacas). On average, the animals were 7 years old, with an average weight of 68 kg for the alpacas and 90 kg for the llamas. During the study, the SAC were kept in a research barn with ad libitum access to water and Coastal Bermuda grass hay, as well as commercially available camelid feed offered twice daily. Study animals were visually examined twice daily by the same veterinarian throughout the study period. All SAC underwent full physical examination before each sampling event and every day throughout the sampling period. A simplified neurologic examination, evaluating the cranial nerves, gait, and cervical motion, was performed on each camelid before each sampling event and once daily for 48 hours after sampling. Behavior and state of consciousness were assessed twice daily at the time of feeding. The centesis sites were examined for swelling, pain, or heat upon palpation.

### 2.2 Study design

The SAC were enrolled in this single‐blinded, randomized, 4‐way, 4‐period crossover study, in which 2 veterinarians each performed both centesis techniques once on each animal. One veterinarian was a second‐year resident in large animal internal medicine (Operator 1) with experience in the LS centesis technique, but limited experience with the AA centesis technique. The second veterinarian was a board‐certified radiologist (Operator 2), who was very experienced with ultrasound‐guided sample collections and had extensive experience with the AA centesis technique in horses. On each of the 4 sampling days, 1 CSF sample was collected from each animal by either AA or LS centesis technique by either operator 1 or 2. Before the study, sampling technique and operator were randomly assigned using an online randomization tool such that each operator and sampling technique combination was performed once in each animal. The first sampling day and the second sampling day occurred 14 days apart. The third sampling day occurred 1 month later, followed by the fourth sampling day 14 days later.

For the CSF collection, SAC were sedated using 1 mL/45 kg body weight (BW) IV of a mixture of anesthetic drugs containing ketamine 83.3 mg/mL (ketamine hydrochloride injection 100 mg/mL, Dechra Veterinary Products, Overland Park, Kansas, USA), xylazine 8.3 mg/mL (AnaSed 100 mg/mL, Akorn Operating Company LLC, Gurnee, Illinois, USA), and butorphanol 0.83 mg/mL (Torbugesic 10 mg/mL, Zoetis US, Parsipanny, New Jersey, USA).^15^ After sedation, SAC were placed in sternal recumbency for CSF collection by either AA or LS approach as described below.

Approximately 2‐3 mL of CSF were collected by gentle syringe aspiration of free‐flowing CSF from the hub of the needle. After sampling, the CSF was transferred into an EDTA blood tube (S‐Monovette, 1.2 mL, Sarstedt, Nümbrecht, Germany) as previously recommended^2^ and submitted for analysis by a board‐certified clinical pathologist blinded to group allocation. All SAC were given 1.1 mg/kg flunixin meglumine IV (Banamine, Merck Animal Health, Madison, New Jersey, USA) after CSF collection.

### 2.3 Lumbosacral centesis technique

The anatomic landmarks were identified by palpation of the dorsal pelvis. The LS space was identified as a palpable depression on midline just cranial to the tuber sacrale. The centesis site was clipped and aseptically prepared using povidone iodine surgical scrub and 70% isopropyl alcohol. While wearing surgical gloves, a 20‐gauge × 3.0 in (76.2 mm) spinal needle (Becton Dickinson, Franklin Lakes, New Jersey, USA) was inserted through the skin perpendicularly to the vertebral column until perceiving tissue resistance or a palpable pop as the needle passed through the interarcuate ligament (*ligamentum flavum*) as described for horses and sheep.^10^, ^16^ Placement of the needle in the subarachnoid space was assessed by withdrawing the stylet of the spinal needle when a sudden movement of the tail was noted (tail swish) as previously described in horses.^10^ If sudden movement or reaction was not observed, needle placement in the subarachnoid space was assessed by intermittently removing the stylet to observe CSF in the needle hub or by wiping the stylet on the back of the sterilely gloved hand. Flow of CSF was enhanced by manually occluding both jugular veins or by gentle aspiration from the needle hub using a 5 mL syringe.

If the operator was confident about having reached the subarachnoid space but CSF flow was not evident, the stylet was reinserted into the needle, and the spinal needle was either withdrawn slightly or reintroduced in a slightly different angle. Either maneuver was considered a repositioning event and was recorded to assess difficulty of sample acquisition. If the needle was fully redrawn from the centesis area and a new needle re‐inserted (second penetration of the skin), it was recorded as a new attempt. If blood was detected in the CSF flow during successful acquisition, the fluid briefly was allowed to flow freely to allow the sample to be clear of iatrogenic blood contamination. If the sample did not become visually clearer, additional collection attempts were not made, and the sample was recorded as blood‐contaminated.

### 2.4 Atlantoaxial centesis technique

**FIGURE 1:**
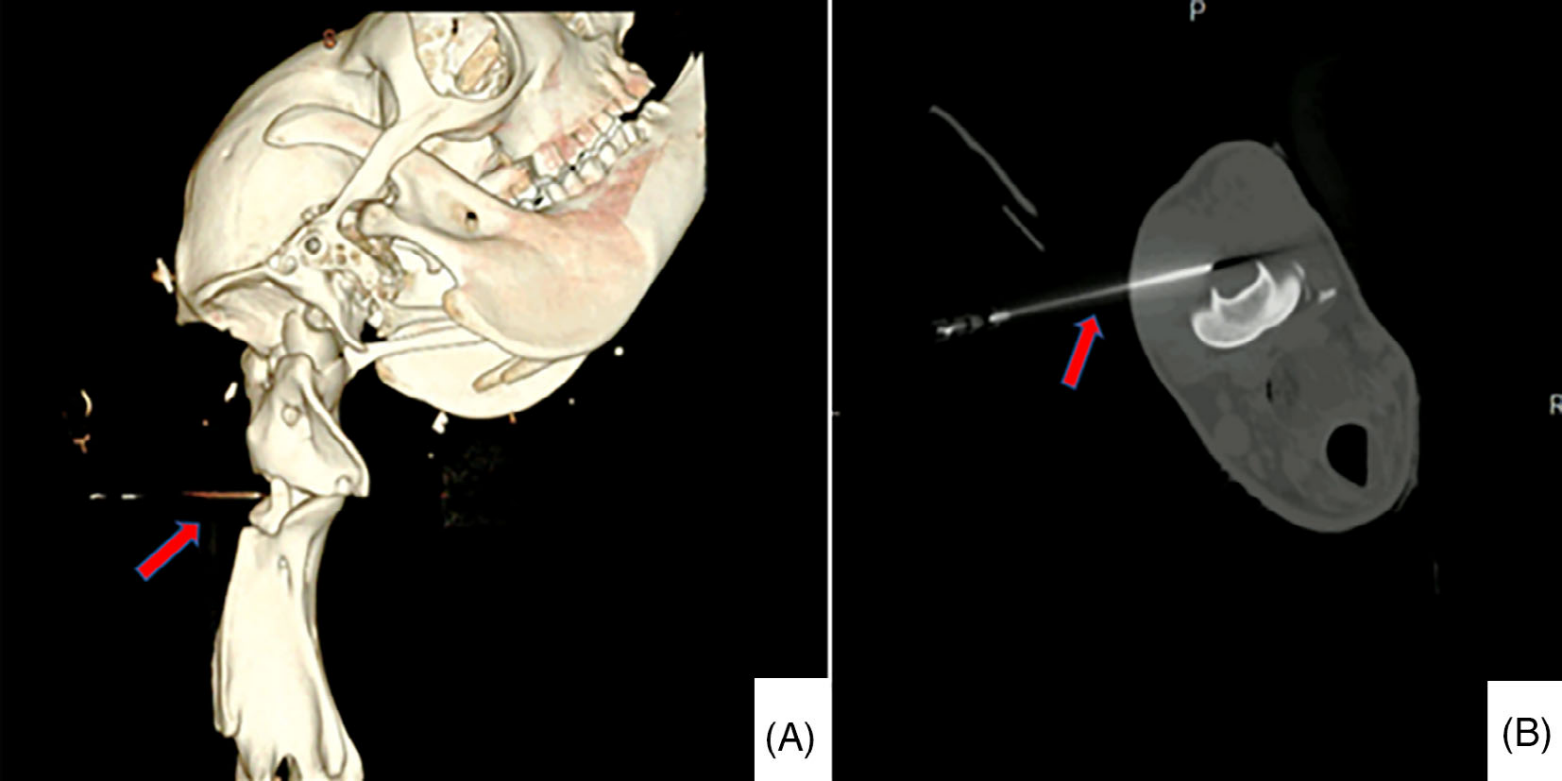
3D reconstruction (A) and transverse (B) computed tomography scan images of the spinal needle in the subarachnoid space during an AA centesis of a SAC carcass. The red arrows point at the spinal needle correctly placed for CSF collection. The spinal needle is inserted in a dorsolateral to ventromedial direction, thus extending axially into the subarachnoid space. The precise angle and insertion depth varies slightly depending on the acoustic window and the individual animal.

After sedation and placement of the animal in sternal recumbency, the neck was placed in slight lateral flexion at an angle of approximately 30°, with the head resting in the assistant's lap. The anatomic landmarks for centesis were located by palpating the transverse process of C1, and then placing the ultrasound probe in a transverse plane just dorsal and caudal to the transverse process of C1 to visualize the accessible subarachnoid space and spinal cord (Figure 1).

**FIGURE 2:**
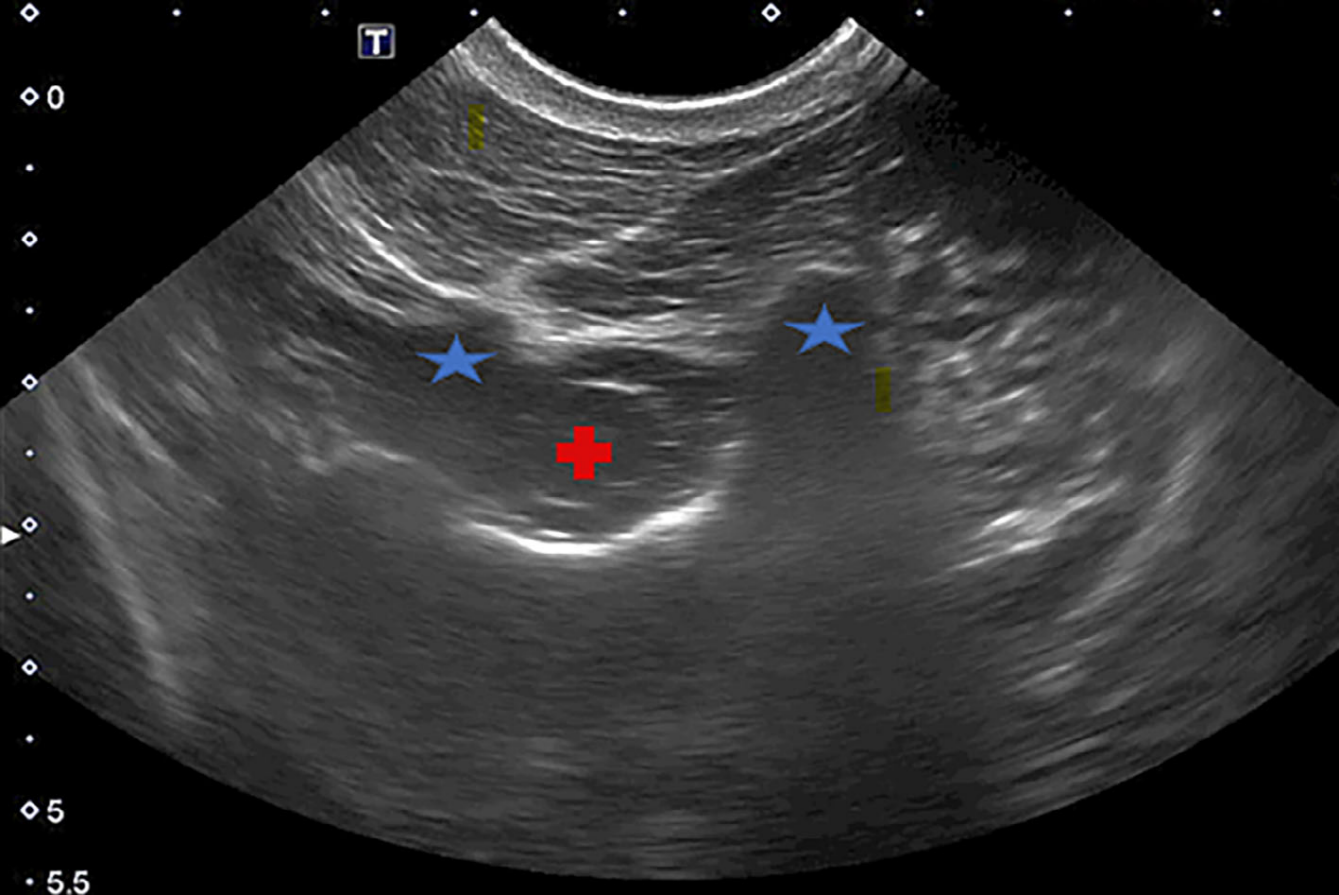
Spinal cord visualized as a hyperechoic oval shape in the middle of the ultrasound screen (red plus sign) and vertebral bodies of C1 and C2 on either side (blue star).

A 20 × 15 cm window was clipped and 70% isopropyl alcohol was applied on the clipped area. The AA space, spinal cord, and surrounding structures were observed ultrasonographically (Sonoscape X5V, SonoScape Medical Corp, Centennial, Colorado, USA) using a 3.0‐5.0 MHz convex probe on a transverse plane. The skin was visualized as the echoic cervical layer covering the hypoechoic muscles. The spinal cord appeared as an anechoic, ovoid structure surrounded by the pia mater, which was visible as a hyperechoic line. Cerebrospinal fluid surrounded the spinal cord within the subarachnoid space. The spinal cord was visualized and the vertebral bodies of C1 and C2 were visualized on either side of the spinal cord (Figure 2), as previously described in horses.^14^, ^17^

After visual confirmation of the correct location for the AA centesis, the skin was prepared aseptically as described for the LS technique. The ultrasound probe was placed in a sterile glove filled with ultrasound transmission gel and the operator donned sterile surgical gloves. A 20‐gauge × 3.0 in (76.2 mm; Becton Dickinson, Franklin Lakes, New Jersey) was used for the aseptic CSF collection. While simultaneously manipulating the ultrasound probe and spinal needle, the operator penetrated the skin and musculature, advancing the needle tip axially into the subarachnoid space surrounding the spinal cord (Figure 2A,B). The needle was consistently visualized by ultrasound, but if it disappeared from sight, slight manipulations of the probe or needle were used to regain visualization. The spinal needle was consistently maintained perpendicularly to the ultrasound beam.

The stylet was maintained in the spinal needle until the dura mater was penetrated. Upon visual confirmation of appropriate needle tip placement in the subarachnoid space, the ultrasound probe was withdrawn, and the stylet was removed from the needle as previously described in horses.^14^ Approximately 2‐3 mL CSF were collected and stored as described for the LS approach. If the operator was confident about reaching the subarachnoid space but CSF flow was not evident, the stylet was reinserted into the needle, and the spinal needle was either withdrawn slightly or reintroduced at a slightly different angle with ultrasonographic visualization. This maneuver was considered a repositioning event and was recorded to assess the difficulty of sample acquisition. If the needle was fully redrawn from the centesis area and a new needle re‐inserted (second penetration of the skin), an additional attempt was recorded.

If blood was detected in the CSF flow during a sample acquisition, the fluid was briefly allowed to flow freely to permit CSF to clear from any iatrogenic blood contamination. If the sample did not become visually clearer, additional sampling attempts were not made, and a visually blood‐contaminated CSF sample was recorded.

### 2.5 Sample analysis

The CSF samples were submitted to the Auburn University, College of Veterinary Medicine clinical pathology laboratory for cytologic analysis within approximately 1 hour of collection, as previously recommended.^2^ Samples were evaluated for color, transparency, total protein concentration (TPC), total nucleated cell count (TNCC), and red blood cell count (RBCC). The TNCC and RBCC were obtained using a manual hemocytometer (Hausser Scientific, Horsham, Pennsylvania, USA). Total protein concentration was determined using a biochemical analyzer (Cobas 311, Roche Diagnostics, Indianapolis, Indiana, USA). A board‐certified clinical pathologist performed cytologic evaluation of the samples and was blinded to the study details and sampling technique used.

### 2.6 Statistical analysis

Age, species, weight, and sex were recorded for each animal. Sampling order, operator, and sampling technique were included in the analysis. The total time (minutes) from initial spinal needle insertion to complete sample acquisition was recorded, as well as the number of attempts and repositioning events. Any reaction (tail or head twitch) by the animal as well as the obtained volume of CSF were recorded.

Commercially available statistical software was used to perform the analyses (GraphPad Software, Boston, Massachusetts, USA and SPSS, IBM, Armonk, New York, USA). Normality of data was determined using the D'Agostino‐Pearson test and visual examination of Q‐Q plots. A repeated measures linear mixed model was built with sampling trial (1‐4), method (AA or LS), and operator (1 or 2) and all interactions of those variables as fixed effects and subject as a random effect for each of the dependent variables: number of attempts, number of repositionings, time, CSF flow (normal or abnormal), reaction (yes or no), RBCC, TNCC, or total protein concentration (TPC). Main effects were compared using the least significant difference (LSD) test. The relationship between RBCC, TNCC, and TPC was analyzed using simple linear regressions. The relationship between order of collection (1‐32) and time was analyzed using simple linear regression. The proportion of animals that had a reaction was compared between methods using McNemar's test. Significance was set at *P* < .05.

## 3 RESULTS

### 3.1 Animals

All SAC recovered from the sedation uneventfully and were standing approximately 15 minutes after CSF centesis. The CSF samples were collected successfully by both operators using either technique. Neither sampling approach resulted in signs of local inflammation or pain, and the animals remained free from neurologic signs. All SAC were released onto the pasture and were considered healthy 3 months after the study was conducted.

### 3.2 CSF analysis

Most of the CSF samples were visually colorless and clear, and 20/32 (63%) were deemed to be unremarkable by the clinical pathologist. Seven of 16 (44%) and 3/16 (19%) of the samples collected by AA and LS centesis, respectively, were observed to be blood contaminated. One sample was observed to be affected by albuminocytological dissociation and another to have lymphocytic pleocytosis, both had been collected by LS centesis from the same animal, on sampling occasions 2 and 3, respectively. On sampling occasion 4, the CSF sample collected from the AA space from the same animal was markedly blood contaminated, and the cytological interpretation therefore could not be performed reliably.

Results of the cytological analysis and comparisons between both centesis techniques are shown in Table 1. The median TNCC was 1/μL (range, 0‐290; 95% confidence interval [CI], −13.4 to 61.3) and 0.5/μL (range, 0‐80; 95% CI, −3.8 to 14.9; *P* = .19) for the AA and LS centesis techniques, respectively. The median RBCC was 167.5/μL (range, 0‐290 000; 95% CI, −16 259 to 56 000) and 155/μL (range, 0‐10 000; 95% CI, −334.2 to 1983; *P* = .25) for the AA and LS technique. The median TPC was 32.9 mg/dL (range, 15.9‐190.8; 95% CI, 15.9‐190.8) and 38 mg/dL (range, 13.4‐120; 95% CI, 33.4‐58.8; *P* = .72) for the AA and LS technique.

**TABLE 1:** Results of the cytological analysis of camelid CSF samples compared between atlantoaxial and lumbosacral centesis techniques.

Cytological variables	AA			LS			*P*‐value
	Median	Range	95% CI	Median	Range	95% CI	
TNC/μL	1	0‐290	0‐61.3	0.5	0‐80	0‐14.9	.19
RBC/μL	167.5	0‐290 000	0‐56 000	155	0‐10 000	0‐1983	.25
TP mg/dL	32.9	15.9‐190.8	19.1‐60.9	38	13.4‐120.5	33.4‐58.8	.72

Abbreviations: AA, atlantoaxial; CI, confidence interval; LS, lumbosacral; RBC, red blood cells; TNC, total nucleated cells; TP, total protein.

The RBCC and TPC results did not differ significantly between serial sampling events with a minimum 2‐week interval (RBCC, *P* = .23; TP, *P* = .79). A significant relationship was observed between serial sampling events and TNCC (*P* = .02). Significant strong correlations were detected between increased RBCC and increased TNCC and TPC (*P* < .001; *R* ^2^ = 0.92 and 0.58, respectively). The relationships between RBCC and TNCC and TPC were determined to be *Y* = 0.001019 × *X* + 4.223 and *Y* = 0.0005170 × *X* + 37.93, respectively.

### 3.3 Feasibility and difficulty of sample collection

Significant differences were not detected when comparing the technical aspects of CSF collection between AA and LS techniques (Table 2). One animal was excluded from the study because of unsuccessful CSF acquisition during the LS centesis technique (marked blood contamination).

**TABLE 2:** Technical parameters associated with the collection of cerebrospinal fluid samples from healthy South American camelids from either the atlantoaxial or lumbosacral sites.

	AA			LS			*P*‐value
	Median	Range	95% CI	Median	Range	95% CI	
Attempts	1	1‐4	0.9‐1.7	1	1‐3	1‐1.6	.82
Repositionings	1	0‐11	0.2‐3.1	0	0‐6	0.1‐1.7	.67
Time (minutes)	2.1	1.2‐17.3	1.2‐8.6	1.3	0.4‐5.6	1.1‐2.3	.16
Reaction	0	0‐1	0‐0.4	1	0‐1	0.3‐0.8	.10

Abbreviations: AA, atlantooccipital; CI, confidence interval; LS, lumbosacral.

Collection of CSF samples resulted in noticeable reactions, characterized as a head twitch in 5/16 (31%) of the animals during the AA approach and a tail swish in 10/16 (63%) of the animals during the LS approach, with no significant differences in the frequency of noticeable reactions between approaches (*P* = .1). The head twitch during AA centesis was observed 3 times during and twice immediately after dura mater puncture. The duration of each event was approximately 2‐5 seconds.

A median of 1 attempt was necessary for both centesis techniques with ranges of 1 to 4 for the AA centesis (95% CI, 0.9‐1.7) and 1‐3 for the LS centesis (95% CI, 1‐1.6; *P* = .82) and the median number of needle repositioning events was 1 for AA (range, 0‐11; 95% CI, 0.2‐3.1) and 0 for LS (range, 0‐6; 95% CI, 0.1‐1.7; *P* = .67). The median time was 2.1 minutes (range, 1.2‐17.3 minutes; 95% CI, 1.2‐8.6) and 1.3 minutes (range, 0.4‐5.6 minutes; 95% CI, 1.1‐2.3) for the AA and LS techniques. The time until successful sample acquisition was not significantly different when comparing both sampling methods (*P* = .16; Table 2).

When comparing the technical aspects of the CSF collection approach between operators, the evaluated main effects did not vary significantly between the novice and experienced operator (Table 3). However, for the number of attempts, significant trial × operator (*P* = .02) and method × operator (*P* = .01) interaction terms were detected, indicating a higher number of attempts was necessary for 1 operator during specific CSF collection trials. No significant correlation (*P* = .14) was found between the order of sample collection (ie, first through 32nd) and time necessary for sample acquisition. The number of repositioning events (*P* = .33) and attempts (*P* = .34) were not related to the order of sample collection for both operators and methods.

**TABLE 3:** Details of technical aspects of CSF sample collections compared between a novice operator (1) and an experienced veterinarian (operator 2) skilled with ultrasound‐guided sample collections.

	Operator 1				Operator 2				
	AA		LS		AA		LS		*P*‐value
	Median	Range	Median	Range	Median	Range	Median	Range	
Attempts	1	1‐4	1	1‐1	1	1‐2	1.5	1‐3	.82
Repositionings	1	0‐11	0	0‐2	1	0‐2	0.5	0‐6	.60
Time (minutes)	3.4	1.2‐17.3	1.2	0.4‐2.4	2.1	1.3‐3.3	1.7	0.5‐5.6	.37
Reaction	0	0‐1	1	0‐1	0	0‐1	0.5	0‐1	.82

Abbreviations: AA, atlantooccipital; LS, lumbosacral.

## 4 DISCUSSION

In our study, CSF samples were collected successfully by either technique during all collection attempts and complications were not observed after repeated sampling. In contrast to our research hypothesis, the AA centesis technique did not yield samples with less blood contamination compared to the LS centesis technique in SAC.

In a previous study in horses, an average of 1.1 attempts was necessary for successful collection of CSF by AA centesis and in another study the median was 1 attempt,^14^, ^18^ which is comparable to the median of 1 attempt for AA centesis in our study. The highest number of attempts and redirections for successful AA centesis, resulting in the longest collection time recorded in our study, occurred with the novice operator attempting the AA centesis technique for the second time, which also was reflected in the significant interaction term identified for the number of attempts. Although it is possible that anatomic conformation of this animal made sample acquisition more difficult,^19^ it was more likely a consequence of the inexperience of the operator because the more experienced operator later performed the AA CSF centesis on this animal without technical difficulties.

In our study, the average time to successfully collect a sample by AA centesis was 3.4 minutes (median, 2.1 minutes), which is similar to previous studies in horses that demonstrated successful collection time to be 2 and 4 minutes.^13^, ^14^ A previous study in horses did not identify a significant difference between median collection times for AA and LS centesis techniques,^13^ which is corroborated by our results. In that same study, both the number of needle redirections and the time of collection for the AA centesis improved as the study progressed and as operator experience increased.^13^ This phenomenon was not observed in our study, in which the necessary repositioning events and attempts for successful sample acquisition were not significantly decreased as the study progressed.

When learning any CSF collection technique, appropriate instruction and sufficient practice of the new technique are necessary to acquire sufficient skills. In other studies, becoming skillful in performing LS centesis decreased the difficulty of sample acquisition and resulted in decreased blood contamination and trauma.^14^, ^20^ Our results suggest that the novel AA centesis technique can successfully be taught to a veterinarian who is skillful with at least 1 CSF centesis technique. Despite acquiring sufficient skills for performing the AA centesis technique, the AA centesis technique could be riskier as compared with LS centesis because of needle placement close to the spinal cord.

In a previous study in horses, 8/15 (53%) of the included healthy animals reacted noticeably to the AA centesis procedure (minor head movement to head jerk), which seemed to be unrelated to dura mater puncture.^13^ As compared to the previous study in horses, fewer animals noticeably reacted to AA centesis in our study. However, in a recently published study in horses, a similar number of horses (4/10) reacted noticeably to AA centesis, unless animals had local anesthesia of the dura mater, muscles, and skin of the AA region, which significantly decreased reactions to AA centesis.^21^ In another study, only 1 of 13 sedated horses reacted noticeably to needle insertion, prompting administration of a local anesthetic.^14^ During LS centesis, 4/15 (27%) of horses in a previous study had a reaction in the form of a tail swish,^13^ which is fewer than observed in our study. It is plausible that variation in utilized sedation and anesthesia protocols influences the occurrence of an observable tail swish and head movement reaction during CSF collection, resulting in differences among studies and species.^13^, ^14^, ^22^ Although commonly utilized as an indicator of correct needle placement, results of our study indicate that reactions such as a tail swish are not always noticeable when performing LS centeses in SAC, indicating that correct needle placement should be verified by other methods as well.

Occasionally, repeated, or serial CSF centesis procedures can be clinically necessary. In a study in horses, cytological changes were not noted in samples obtained by serial collection with a 2‐week interval between collections.^18^ In contrast, a study performed in healthy human volunteers indicated that repeated CSF centesis within a 2‐week interval caused alterations in the TNCC of the collected samples,^23^ which is similar to our study. Repeated sampling may have induced an inflammatory response in the CNS, which could affect the TNCC of the CSF.

In contrast to a study in horses, in which mild swelling and sensitivity at the centesis site were reported after AA centesis,^13^ SAC in our study were not affected by pain or swelling at either centesis site. Similar to results from our study, horses in another report did not show signs of adverse effects within 48 hours after AA centesis.^14^ A recent study in horses described transient hyperthermia after AA centesis, regardless of whether or not local analgesia was administered before centesis.^21^ Necropsy examination of horses after AA centesis documented occurrence of subarachnoid hemorrhage, edema and hemorrhage of the neck musculature and the cervical epidural soft tissues, and axonal degeneration.^13^, ^24^ Because the AA centesis technique is a novel procedure in SAC, reports of adverse effects are currently not available. It is plausible that similar complications may occur in SAC, and choosing the AA technique therefore should be governed by clinical circumstances, neuroanatomical localization of disease, operator experience, animal compliance, animal anatomy, and availability of necessary equipment.^9^, ^17^

Iatrogenic blood contamination of CSF can result from operator inexperience, anatomic conformation of the animal, variations in vascular anatomy, or presence of underlying disorders that negatively affect sample acquisition.^19^ The use of ultrasonography during AA and AO centesis decreased blood contamination, compared with blinded centesis from the LS space in large animals.^7^, ^9^, ^13^ In contrast to these studies, we did not identify a significant difference between the RBCC in samples collected by the ultrasound‐guided AA technique with those collected by the blind LS technique.

A limitation of our study was the small sample size of 8 SAC, which was governed by the availability of animals in the university teaching herd. Including additional animals would have improved statistical power, but most of the results were very similar (eg, median attempts 1 vs 1), and detecting significant differences may require very large sample sizes. Another important limitation of our study was the inclusion of only healthy individuals, and future research should evaluate the novel AA centesis technique in clinical settings.

## 5 CONCLUSION

Our results suggest that the ultrasound‐guided AA centesis technique does not yield samples with less blood contamination as compared with the blinded LS centesis technique. Both centesis techniques were comparable regarding the technical aspects of collection. Our results indicate that the AA technique could be an acceptable alternative to other CSF collection techniques in SAC, as governed by clinical circumstances, neuroanatomical localization of disease, operator experience, animal compliance, animal anatomy, and availability of necessary equipment. Additional research should be performed to evaluate ultrasound‐guided AA centesis in animals with neurological disease.

## CONFLICT OF INTEREST DECLARATION

Authors declare no conflict of interest.

## OFF‐LABEL ANTIMICROBIAL DECLARATION

Authors declare no off‐label use of antimicrobials.

## INSTITUTIONAL ANIMAL CARE AND USE COMMITTEE (IACUC) OR OTHER APPROVAL DECLARATION

Approved by the Auburn University IACUC, PRN 2021‐3837.

## HUMAN ETHICS APPROVAL DECLARATION

Authors declare human ethics approval was not needed for this study.
